# Quadriceps Angle Measurement in Adolescents With Short Stature: Exploring the Relationship Between Postural Alignment and Lower Limb Mechanics

**DOI:** 10.7759/cureus.43953

**Published:** 2023-08-23

**Authors:** Rahul Sharma, Gitanjali Khorwal, Vikas Vaibhav, Brijendra Singh, Raviprakash Meshram

**Affiliations:** 1 Anatomy, All India Institute of Medical Sciences, Rishikesh, Rishikesh, IND; 2 Forensic Medicine and Toxicology, All India Institute of Medical Sciences, Rishikesh, Rishikesh, IND; 3 Forensic Medicine, All India Institute of Medical Sciences, Rishikesh, Rishikesh, IND

**Keywords:** quadriceps angle (q- angle), adolescents with short stature, children and adolescents, q-angle, quadriceps angle, genu varum, chondromalacia patella, patellofemoral pain, anthropometry, short stature

## Abstract

Background

The quadriceps angle (Q angle) is measured as an angle formed by two lines that extend from the anterior superior iliac spine to the midpoint of the patella and from the midpoint of the patella to the tibial tuberosity. The average Q angle value for children aged between seven to 12 years was 13.1˚±3.5˚in boys and 13.7˚±4.9˚ in girls, whereas 8˚-15˚ in men and 12˚-19˚ in women. Abnormal variation in Q angle is associated with patellofemoral pain syndrome, lateral patellar malposition, dislocation, chondromalacia patella, patella alta, genu varum, etc.

Methodology

The present study explores the status of Q angle values among adolescents with short stature and their comparison with age and gender-matched children between 10 and 15 years of age.

Results

We found a statistically significant difference between the Q angle value in the control group and the male with short stature group aged 14-15 years, with a mean difference of 3.7˚. However, among females, there was a significant difference between the control group and the short-stature group aged 12-13 and 14-15 years, with a mean difference of 2.8˚ and 2.5˚, respectively.

Implications

Early detection and timely remedial measures, e.g., quadriceps strengthening exercises, before skeleton maturity can prevent Q angle-related misalignments and abnormalities in the limb.

## Introduction

Based on obtainable articles, the quadriceps angle (Q angle) was defined for the first time by Brattstroem in 1964, who determined it as an angle between the ligamentum patella and the extension of the line depicting the net resultant force of quadriceps femoris muscles acting on the patella [1]. Therefore, the Q angle of the knee is principally a measurement of the angle between the quadriceps muscles and the patella tendon. However, at present, the anterior superior iliac spine (ASIS) is used as a convenient and easily discernible proximal landmark for the measurement [2]. The Q angle provides useful information about the position of the knee joint and is hence an essential indicator of the biomechanical function of the lower extremity. Values on either side of the normal range are seen to be associated with pathological conditions (e.g., chondromalacia and recurrent dislocation of the patella), gait abnormalities, and increased susceptibility to injuries [3].

The average Q angle value for children aged between seven to 12 years was 13.1±3.5˚ in boys and 13.7±4.9˚ in girls. The Q angle is marginally greater in females than in males because of the wider pelvis [4]. The average Q angle value for adults, when measured in standing, was 8˚-15˚ in men and 12˚-19˚ in women [5].

Studies show that any abnormal variation from the normal value of the Q angle is associated with various pathologies of the lower extremities. An increase in Q angle to 15˚-20˚ may cause lateral patellofemoral contact pressures that may lead to patellofemoral pain syndrome (PFPS) with lateral patellar malposition (responsible for early degeneration of adjacent joint capsule) or dislocations; dysfunction of the knee extensor mechanism with femoral ante-version, external tibial torsion, laterally displaced tibial tubercle, or genu valgum [5-8]. Although a decreased Q angle may not cause a medial shift to the patella, it may increase the medial tibiofemoral contact pressure between the medial femoral and tibial condyles by increasing the varus orientation. Abnormally low values of Q angle are also likely to develop chondromalacia patella, patella alta, and genu varum [9].

These variations in Q angle likely occur because of the relative mobility caused by the pull of the patellar tendon over the knee joint. Any disturbance in the net lateral force on the patella due to the contraction of the quadriceps muscle results in knee extensor mechanism malalignment, causing hypermobility and patellar instability at the knee joint. With an increase in regional stresses, they ultimately cause uni-compartmental osteoarthritis earlier compared with the normal population [10].

Despite the crucial role of the Q angle on lower extremity alignment and functionality, we could not find such studies performed on subjects with short stature. Short stature is defined as a condition in which an individual's height is inside the third percentile for the mean height of a given age, sex, and population group [11]. The present study was designed in Rishikesh, Uttarakhand, India, to find out the Q angle value in adolescent children with short stature and its variation among boys and girls with short stature compared to those who fall in the normal spectrum.

## Materials and methods

Subsequent to receiving the required approval from the Institutional Ethics Committee of the All India Institute of Medical Sciences, Rishikesh (Uttarakhand), reference IEC/21/302 dated May 15, 2021, and after explaining the study briefly to the school principals and obtaining written consent and approval, 90 (45 of each gender) children with short-stature were selected for the study.

Children with a history of knee surgery, neurological deficit, open wound, cancer or tumor, any orthopedic or metabolic disease involving the lower extremities, and any systemic or localized infection were excluded.

Following preliminary considerations, children between 10-15 years of age whose height was equal to or less than given in the third percentile category in the Data Table of Stature-for-age Charts for males and females aged two-20 years issued by the National Center for Health Statistics and retrieved from the website of the Centers for Disease Control and Prevention, U.S. Department of Health & Human Services, were included in the study given (Appendices A-B) [12]. A control group consisting of 90 children of matched age and gender with normal height was also selected to measure the bilateral Q angle. Written informed consent was also obtained by the principals of the schools and the students after thoroughly explaining the procedure for measuring the Q angle.

It has been established that goniometry for measuring Q angle can be used as an inexpensive and radiation-free alternative to radiographic measurement [13]. In order to get information on the alignment of the knee in the frontal plane, taking into account typical weight-bearing loads, the measurements were done with the subject in a standing position.

**Figure 1:**
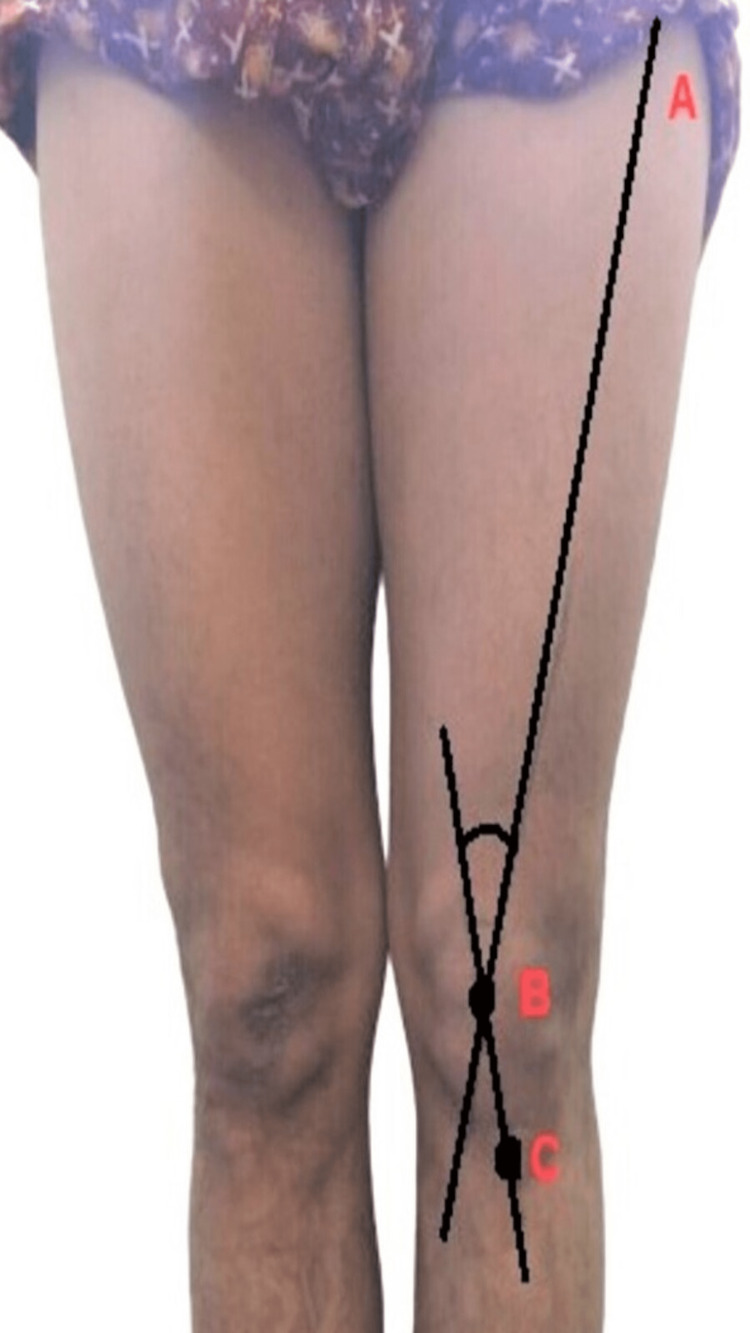
Reference points to measure the Q angle: A) anterior superior iliac spine (ASIS); B) midpoint of the patella; C) tibial tuberosity

Therefore, for measuring the Q angle, the participants were asked to stand in the anatomical position (standing erect, shoulders held back with fully extended elbows, and forearms fully supinated). The reference points, viz., anterior superior iliac spine (ASIS, point A), mid-point of the patella (point B), and tibial tuberosity (point C), were palpated and marked by using a black marker pen. Two straight lines were drawn from points A to B and point B to C; the angle formed between these two lines was measured as a Q angle, as shown in Figure 1.

**Figure 2:**
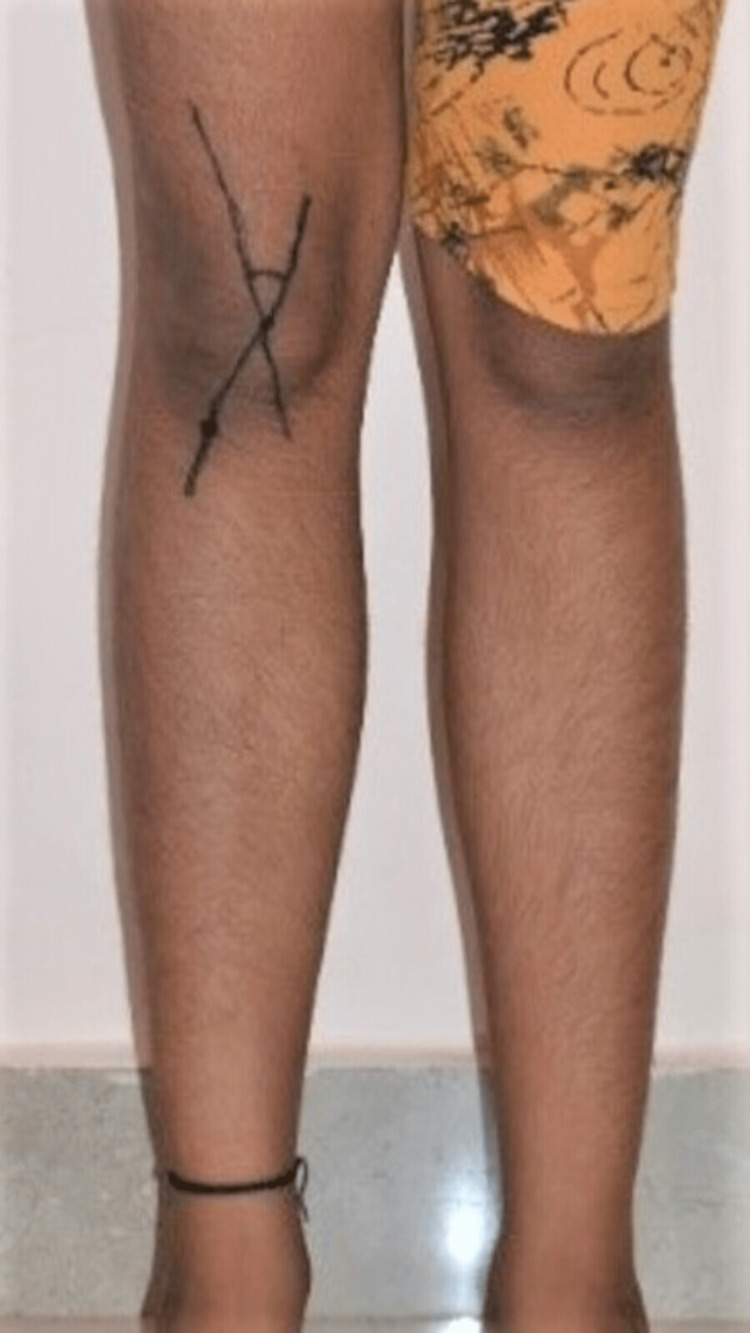
Markings on the subject’s knee

**Figure 3:**
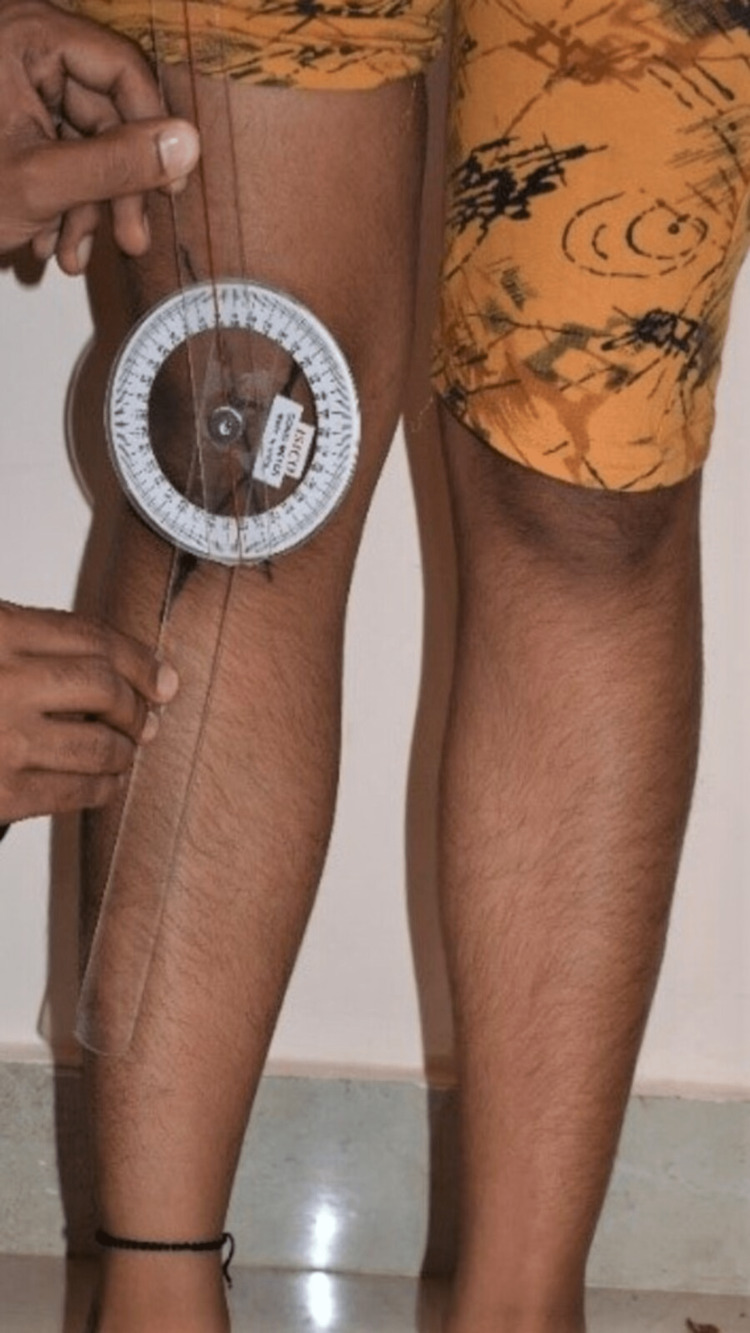
Measuring the Q angle using a standard goniometer (ISICO goniometer)

The angle formed (Figure 2) was measured using a standard goniometer (Figure 3) in both limbs, and the results were tabulated for both control and short stature groups for mean values.

The data collected were statistically analyzed using IBM SPSS software version 22 (IBM Corp., Armonk, NY, USA) with a 0.05 (5%) significance level.

## Results

Children with short and normal statures were included in the study (Tables 1-2).

**Table 1:** The average height of male and female children with short stature cm: centimeters

Years	Total no. of children with short stature	Average height of males with short stature (cm)	Average height of females with short stature (cm)
10-11	21	130.54	131.01
12-13	39	135.92	137.94
14-15	30	143.63	146.08

**Table 2:** The average height of male and female children with normal stature cm: centimeters

Years	Total no. of children with normal stature	Average height of males with normal stature (cm)	Average height of females with normal stature (cm)
10-11	21	133.53	137.38
12-13	39	137.79	149.45
14-15	30	160.32	151.94

Measurements of the Q angle and range for children with short stature aged between 10 and 15 years were obtained by calculating the mean and standard deviation for each gender group. The mean Q angle in short-statured boys was 9.5˚±1.4˚ while in short-statured girls it was 10˚±1.6˚. The mean Q angle for all 180 legs in 90 children was 9.7˚±1.5˚ (Table 3).

**Table 3:** Measurement of the Q angle for boys and girls with short stature

Gender	No of children	No of legs	Q angle (Mean ± SD)	Range	p-value
Male	45	90	9.5˚±1.4˚	7˚-14˚	0.468
Female	45	90	10˚±1.6˚	8˚-14˚	
Total	90	180	9.7˚ ±1.5˚	7˚ -14˚	

There was no statistically significant difference between the Q angles measured in both genders (p-value = 0.468).

There was no statistically significant difference in the Q angles of the right and left knees in both groups (Table 4).

**Table 4:** Measurement of the Q angle in the right and left knees in short-statured children

	Male	Female	p-value
Q angle (right)	9.64˚ ±1.554˚	10.00˚ ±1.651˚	0.41
Q angle (left)	9.42˚ ±1.373˚	10.02˚ ±1.644˚	0.23

A three-way analysis of variance (ANOVA) was used between the age groups to evaluate the correlation between the Q angle values and age in both male and female children with short stature. The test values show there was a significant correlation between the two, i.e., as age increased, there was an increase in Q angle in both the girl's and boys' short-stature groups.

The Q angle measure in boys with short stature, aged 10-11 years, was 8.9˚±1.1˚; for ages 12-13 years, the values were 9.6˚±1˚; and for ages 14-15 years, the values of the Q angle were 10.2˚±1.4˚ (Table 5).

**Table 5:** Comparison of the Q angle among each age group of boys with short stature

Age (years)	Mean Q angle	SD	Range	p-value
10-11	8.9˚	1.1˚	7-13	0.0023
12-13	9.6˚	1˚	7-12	
14-15	10.2˚	1.4˚	8-14	

The Q angle measure in girls with short stature, for those aged between 10-11 years, was 9.1˚±1˚; for ages 12-13 years, the values were 9.8˚±1.5˚ degrees; and for ages 14-15 years, the values of Q angle were 10.7˚±1.8˚ (Table 6).

**Table 6:** Comparison of the Q angle among each age group of girls with short-stature

Age (years)	Mean Q angle	SD	Range	p-value
10-11	9.1 ˚	1˚	8-12	0.001
12-13	9.8 ˚	1.5 ˚	8-14	
14-15	10.7 ˚	1.8 ˚	8-13	

A comparison of male Q angle values between the control group and the short stature group showed a significant difference in the age groups of 10-11 years, 12-13 years, and 14-15 years, with a mean difference of 3˚, 3.3˚, and 3.7˚, respectively (Table 7).

**Table 7:** Comparison of the Q angle between the control group and the group of males with short stature in different age groups

Age (years)	Control	Short stature	Mean diff	p-value
10-11	11.9˚±1.09˚	8.9˚±1.1˚	3˚	< 0.0001
12-13	12.9˚± 1˚	9.6˚±1˚	3.3˚	<0.0001
14-15	13.8˚±0.7˚	10.5˚±1.8˚	3.7˚	0.0009

Comparison of female Q angle values between the control group and the short stature group showed a significant difference in the age groups of 10-11 years, 12-13 years, and 14-15 years, with a mean difference of 3.2˚, 2.8˚, and 2.5˚, respectively (Table 8).

**Table 8:** Comparison of the Q angle between the control group and the group of females with short stature in different age groups

Age (years)	Control	Short stature	Mean diff	p-value
10-11	12.3˚±1.1˚	9.05˚±1˚	3.2˚	0.0001
12-13	12.6˚ ±1.04˚	9.8˚±1.5˚	2.8˚	0.02
14-15	13.2˚± 0.8˚	10.7˚±1.8˚	2.5˚	0.00004

Based on the above results and trends, we can say that among children with short stature, the difference in the Q angle compared to the normal population is statistically significant (p-value<0.05).

The results show no significant relationship in the Q angle value between the right and left knee in both females and males with short stature. The mean Q angle value for all 180 legs in children with short stature aged 10-15 years was 9.7˚±1.5˚. Whereas, the mean Q angles for males and females with short stature were 9.5˚±1.4˚ and 10˚±1.5˚, respectively.

Whereas there is a positive correlation between age and Q angle, i.e., with the increase in age, there is an increase in Q angle. The average Q angle value in boys aged 10-11 years was 8.9˚±1.1˚ and 9.1˚±1˚in girls, and for ages 12-13, it was 9.6˚±1˚ in boys and 9.8˚±1.5˚ in girls, whereas, for ages 14-15, the Q angle value in the boys was 10.2˚±1.4˚ and 10.7˚±1.8˚ in girls.

## Discussion

The present study was conducted to measure the quadriceps angle in adolescent girls and boys with short stature. Results show that the mean Q angle value for all 180 legs in children with short stature aged between seven to 14 years was 9.7˚±1.5˚. The mean Q angles for males and females with short statures were 9.5˚±1.4˚ and 10˚±1.6˚, respectively.

Cankaya et al. conducted a study in which they assessed the quadriceps angle in children aged two to eight years and gave an average Q angle in standing position of 13.27˚±1.22°for the right leg in boys and 13.30˚±1.16°for girls. The mean Q angle for the left leg in boys was 13.25˚±1.23° and for girls, it was 13.29˚±1.18°. The average Q angle in the supine position for the right leg in boys was 13.30˚± 1.21° and 13.32˚± 1.17˚ for girls; the mean Q angle for the left leg in boys was 13.25˚±1.22°, and for girls, it was 13.29˚±1.14° [5]. The Q angle for children between the ages of two to four years in the standing position, measured in the right leg, was 14.10˚±0.67°and for the left leg, it was 14.07˚±0.69°. For children aged four to six years, the Q angle for the right leg was 13.08˚±1.41° and for the left leg, it was 13.06˚±1.43° and lastly, the Q angle for children aged six to eight years, for the right leg, was 12.71˚±0.87° and for the left leg, it was 12.69˚±0.90° and found that in all age groups, no significant differences were found between the right and left Q angle values in all age groups in both sex groups, which means girls’ mean Q angle values and the boys’ mean Q angle values were similar. This study result is in accordance with our study, as we found no significant difference between right and left Q angle values in both sexes in children with short stature.

We found no significant difference in the Q angle value between boys and girls with short stature. This is consistent with that of Bhalara et al., who measured the Q angle in children aged between seven to 12 years and found that the Q angle in the normal population was 15.7˚±4˚ in boys and 15.8˚±3.4˚ in girls [1]. They also found no comparative disparity in the Q angle value among both genders. Using a three-way ANOVA between subgroups, they concluded that as age increases, there is a significant increase in Q angle among children. The average Q angle value in boys for ages seven to eight years was 10.1˚±0.9˚ and 12.5˚±2.6˚in girls, and for ages nine to 10, it was 15.6˚±4.5˚ in boys and 15.7˚±3.4˚ in girls, whereas, for ages 11-12, the Q angle value in the boys was 18.3˚±3.3˚ and 16.8˚±3˚ in girls. This is in accordance with our study, as we also found a positive correlation between age and Q angle.

The mean Q angle value for all 180 legs in children with short stature aged 10-15 years was 9.7˚±1.5˚. Whereas, the mean Q angles for boys and girls with short stature were 9.5˚±1.4˚ and 10˚±1.6˚, respectively. The average Q angle value in boys for ages 10-11 years was 8.9˚±1.1˚ and 9.1˚± 1˚in girls, and for ages 12-13, it was 9.6˚±1˚ in boys and 9.8˚±1.5˚ in girls, whereas, for ages 14-15, the Q angle value in the boys was 10.2˚±1.4˚ and 10.7˚±1.8˚ in girls. These values for normal subjects are different from those mentioned in the study performed by Khasawneh et al. on 500 volunteers, a young Arab population [14]. They found that the average mean Q angle in the females was 17.35˚±0.225˚ and that in the males was 14.1˚± 0.21˚. The Q angle values in both genders of the Arab population were relatively higher than in other countries and ethnicities. Compared to our study, it may be attributed to racial, ethnic, and probably socio-economic differences among Indian and Arab populations.

The quadriceps angle is an important tool to determine knee health. However, the value of the Q angle for children with normal height has varied, as reported by different researchers in the literature [15].

However, comparing the Q angle values of children with short stature with age and gender-matched control groups showed statistically significant differences among the age groups of 10-11 years, 12-13 years, and 14-15 years in both genders (p-value > 0.05).

However, there were some limitations to our study; it was conducted during the COVID-19 pandemic, and many of the classes were suspended in the schools in Rishikesh where the study was conducted. The area of our study was limited to Rishikesh. The population with short stature comprises 1/3 of the population with normal stature; that’s why the sample size is too small, and further studies should be conducted over a larger area with a larger sample size.

## Conclusions

The quadriceps angle is an important tool to determine knee health. However, the value of the Q angle for children with normal height has varied, as reported by different researchers in the literature. But there is very little or no literature that evaluates quadriceps angles in children with short stature or has been accepted by clinicians. In our study done among children with short stature, results showed that the mean Q angle value for all 180 legs in children with short stature aged seven to 14 years was 9.7˚±1.5˚. The mean Q angles for boys and girls with short statures were 9.5˚±1.4˚ and 10˚±1.6˚, respectively. Therefore, by knowing the normal range and values of the Q angle, clinicians can properly define and identify the abnormal range and value of the quadriceps angle in the population of children with short stature.

Early detection of Q angle variation in younger populations and resting Q angle value may enhance the clinician's ability to predict which individuals are at significant risk of developing patellar tracking and patellofemoral disease that worsens with older age.

Different remedial measures, e.g., quadriceps strengthening exercises, can be instituted by clinicians to correct the abnormality at an early age before reaching skeleton maturity.

## Appendices

Appendix A 

**Table 9:** Data table of stature-for-age charts (males, age 2–20 years) as per CDC guidelines [12]

Age (in months)	3rd Percentile Stature (in centimeters)	5th Percentile Stature (in centimeters)	10th Percentile Stature (in centimeters)	25th Percentile Stature (in centimeters)	50th Percentile Stature (in centimeters)	75th Percentile Stature (in centimeters)	90th Percentile Stature (in centimeters)	95th Percentile Stature (in centimeters)	97th Percentile Stature (in centimeters)
24	79.91084	80.72977	81.99171	84.10289	86.4522	88.80525	90.92619	92.19688	93.02265
24.5	80.26037	81.08868	82.36401	84.49471	86.86161	89.22805	91.35753	92.63177	93.45923
25.5	81.00529	81.83445	83.11387	85.25888	87.65247	90.05675	92.22966	93.53407	94.38278
26.5	81.73416	82.56406	83.84716	86.00517	88.42326	90.8626	93.07608	94.40885	95.27762
27.5	82.44846	83.27899	84.56534	86.73507	89.17549	91.64711	93.89827	95.25754	96.14512
28.5	83.14945	83.98045	85.26962	87.44977	89.91041	92.41159	94.69757	96.08149	96.98663
29.5	83.83819	84.66948	85.96098	88.15028	90.62908	93.15719	95.47522	96.88198	97.80345
30.5	84.51558	85.34694	86.64027	88.83745	91.33242	93.88496	96.23239	97.66027	98.59691
31.5	85.18238	86.01357	87.3082	89.51202	92.02127	94.59585	96.97022	98.41758	99.36828
32.5	85.83925	86.66999	87.9654	90.17464	92.69638	95.2908	97.68978	99.15514	100.1189
33.5	86.48678	87.3168	88.61244	90.82592	93.35847	95.97068	98.39218	99.87416	100.8501
34.5	87.12552	87.95452	89.24986	91.46645	94.00823	96.63637	99.07848	100.5759	101.5631
35.5	87.75597	88.58366	89.87816	92.0968	94.64637	97.28875	99.74979	101.2615	102.2593
36.5	88.37864	89.20473	90.49789	92.71756	95.27359	97.9287	100.4072	101.9324	102.9402
37.5	88.93297	89.77301	91.08608	93.3344	95.91475	98.58525	101.069	102.593	103.5983
38.5	89.47916	90.33306	91.66589	93.94268	96.54734	99.23358	101.7234	103.247	104.2503
39.5	90.01766	90.88532	92.23779	94.54291	97.17191	99.87426	102.3709	103.8948	104.8967
40.5	90.54891	91.43025	92.80225	95.13557	97.78898	100.5078	103.012	104.537	105.538
41.5	91.07337	91.96832	93.35972	95.72115	98.39903	101.1348	103.6473	105.1739	106.1747
42.5	91.59152	92.49999	93.91068	96.30009	99.00254	101.7556	104.2771	105.8061	106.8071
43.5	92.10382	93.0257	94.45556	96.87286	99.59998	102.3708	104.9021	106.434	107.4357
44.5	92.61073	93.54592	94.99482	97.43989	100.1918	102.9807	105.5225	107.0579	108.0609
45.5	93.11271	94.06109	95.52888	98.00159	100.7783	103.5858	106.1387	107.6784	108.683
46.5	93.61022	94.57166	96.05817	98.55838	101.36	104.1865	106.7513	108.2956	109.3024
47.5	94.10371	95.07806	96.5831	99.11064	101.9373	104.7831	107.3604	108.9101	109.9193
48.5	94.59361	95.5807	97.10407	99.65875	102.5105	105.3759	107.9665	109.522	110.5342
49.5	95.08035	96.08	97.62147	100.2031	103.0799	105.9654	108.5698	110.1317	111.1473
50.5	95.56435	96.57635	98.13566	100.7439	103.6459	106.5518	109.1706	110.7394	111.7588
51.5	96.046	97.07013	98.64701	101.2817	104.2087	107.1354	109.7693	111.3454	112.369
52.5	96.52568	97.5617	99.15585	101.8166	104.7687	107.7165	110.366	111.95	112.9781
53.5	97.00376	98.05141	99.6625	102.3491	105.3262	108.2953	110.9609	112.5533	113.5863
54.5	97.48058	98.53958	100.1673	102.8792	105.8813	108.872	111.5543	113.1555	114.1937
55.5	97.95648	99.02654	100.6705	103.4074	106.4343	109.4469	112.1464	113.7568	114.8006
56.5	98.43175	99.51256	101.1723	103.9339	106.9855	110.0201	112.7374	114.3574	115.4072
57.5	98.90667	99.99791	101.6731	104.4588	107.535	110.5919	113.3273	114.9575	116.0134
58.5	99.38151	100.4828	102.173	104.9825	108.083	111.1623	113.9164	115.557	116.6194
59.5	99.8565	100.9676	102.6723	105.505	108.6296	111.7316	114.5047	116.1561	117.2254
60.5	100.3318	101.4523	103.1712	106.0265	109.1751	112.2998	115.0924	116.755	117.8314
61.5	100.8077	101.9372	103.6697	106.5472	109.7196	112.8671	115.6795	117.3536	118.4374
62.5	101.2843	102.4225	104.1682	107.0673	110.2631	113.4335	116.2661	117.9521	119.0435
63.5	101.7618	102.9082	104.6666	107.5868	110.8058	113.9992	116.8522	118.5505	119.6498
64.5	102.2401	103.3945	105.1651	108.1058	111.3477	114.5641	117.438	119.1487	120.2562
65.5	102.7195	103.8814	105.6638	108.6244	111.889	115.1284	118.0234	119.7469	120.8627
66.5	103.2	104.369	106.1627	109.1427	112.4296	115.6921	118.6084	120.345	121.4694
67.5	103.6815	104.8574	106.6619	109.6607	112.9696	116.2551	119.1931	120.943	122.0761
68.5	104.1642	105.3466	107.1614	110.1785	113.509	116.8176	119.7774	121.5408	122.6829
69.5	104.6479	105.8364	107.6611	110.696	114.0479	117.3794	120.3613	122.1384	123.2897
70.5	105.1326	106.327	108.1612	111.2132	114.5861	117.9407	120.9447	122.7359	123.8965
71.5	105.6183	106.8182	108.6614	111.7302	115.1238	118.5012	121.5277	123.333	124.5031
72.5	106.1048	107.3099	109.1619	112.2469	115.6609	119.0611	122.1101	123.9297	125.1095
73.5	106.5921	107.8021	109.6624	112.7631	116.1973	119.6203	122.6918	124.526	125.7156
74.5	107.0799	108.2946	110.1629	113.2789	116.7329	120.1786	123.2729	125.1217	126.3212
75.5	107.5682	108.7873	110.6633	113.7942	117.2678	120.7361	123.8532	125.7168	126.9263
76.5	108.0566	109.2801	111.1634	114.3089	117.8018	121.2926	124.4327	126.3111	127.5307
77.5	108.5451	109.7727	111.6631	114.8229	118.3348	121.848	125.0111	126.9045	128.1344
78.5	109.0335	110.2649	112.1623	115.336	118.8668	122.4024	125.5884	127.4969	128.7371
79.5	109.5214	110.7566	112.6608	115.8481	119.3977	122.9555	126.1646	128.0882	129.3387
80.5	110.0086	111.2476	113.1583	116.3592	119.9272	123.5073	126.7394	128.6782	129.9391
81.5	110.495	111.7375	113.6548	116.869	120.4554	124.0576	127.3128	129.2668	130.5381
82.5	110.9801	112.2263	114.1499	117.3774	120.9821	124.6064	127.8846	129.8538	131.1356
83.5	111.4638	112.7135	114.6436	117.8842	121.5072	125.1535	128.4547	130.4392	131.7314
84.5	111.9459	113.1991	115.1356	118.3893	122.0305	125.6987	129.023	131.0226	132.3253
85.5	112.4259	113.6827	115.6257	118.8926	122.552	126.2421	129.5893	131.6041	132.9172
86.5	112.9036	114.1642	116.1136	119.3938	123.0714	126.7834	130.1535	132.1834	133.507
87.5	113.3789	114.6431	116.5992	119.8927	123.5886	127.3225	130.7154	132.7605	134.0943
88.5	113.8513	115.1194	117.0822	120.3893	124.1035	127.8594	131.275	133.335	134.6792
89.5	114.3206	115.5927	117.5625	120.8833	124.616	128.3937	131.8321	133.907	135.2615
90.5	114.7867	116.0629	118.0398	121.3746	125.1259	128.9256	132.3865	134.4763	135.8409
91.5	115.2491	116.5297	118.5139	121.863	125.6331	129.4547	132.9381	135.0426	136.4173
92.5	115.7077	116.9928	118.9847	122.3483	126.1374	129.981	133.4868	135.606	136.9906
93.5	116.1623	117.4521	119.4519	122.8305	126.6388	130.5044	134.0325	136.1662	137.5607
94.5	116.6127	117.9074	119.9153	123.3092	127.137	131.0247	134.5751	136.7231	138.1274
95.5	117.0587	118.3585	120.3749	123.7845	127.632	131.5419	135.1144	137.2767	138.6906
96.5	117.5	118.8053	120.8305	124.2562	128.1237	132.0559	135.6504	137.8267	139.2502
97.5	117.9366	119.2475	121.2819	124.7242	128.6119	132.5664	136.1829	138.3731	139.806
98.5	118.3683	119.6851	121.729	125.1882	129.0966	133.0736	136.7118	138.9159	140.358
99.5	118.7949	120.1179	122.1716	125.6484	129.5777	133.5771	137.2371	139.4548	140.9062
100.5	119.2165	120.5459	122.6099	126.1045	130.055	134.0771	137.7587	139.9899	141.4503
101.5	119.633	120.969	123.0435	126.5565	130.5286	134.5734	138.2765	140.5211	141.9904
102.5	120.0442	121.3872	123.4726	127.0044	130.9983	135.066	138.7905	141.0484	142.5263
103.5	120.4502	121.8004	123.897	127.4481	131.4641	135.5548	139.3006	141.5716	143.0582
104.5	120.851	122.2086	124.3168	127.8876	131.926	136.0397	139.8069	142.0908	143.586
105.5	121.2467	122.6119	124.7319	128.3228	132.384	136.5209	140.3093	142.6061	144.1096
106.5	121.6372	123.0103	125.1425	128.7539	132.8381	136.9982	140.8077	143.1173	144.6291
107.5	122.0228	123.4039	125.5485	129.1807	133.2882	137.4717	141.3023	143.6245	145.1445
108.5	122.4034	123.7928	125.9501	129.6035	133.7345	137.9414	141.793	144.1278	145.656
109.5	122.7793	124.1771	126.3473	130.0222	134.1769	138.4073	142.28	144.6272	146.1634
110.5	123.1506	124.5569	126.7402	130.4369	134.6155	138.8696	142.7632	145.1228	146.6671
111.5	123.5175	124.9325	127.1291	130.8477	135.0504	139.3282	143.2428	145.6148	147.167
112.5	123.8803	125.304	127.514	131.2548	135.4818	139.7833	143.7188	146.1032	147.6633
113.5	124.2391	125.6717	127.8953	131.6584	135.9097	140.235	144.1915	146.5882	148.1562
114.5	124.5943	126.0358	128.273	132.0585	136.3343	140.6835	144.661	147.0699	148.6459
115.5	124.9462	126.3966	128.6474	132.4555	136.7557	141.1289	145.1273	147.5486	149.1325
116.5	125.295	126.7544	129.0189	132.8495	137.1742	141.5713	145.5909	148.0245	149.6163
117.5	125.6413	127.1096	129.3876	133.2407	137.5899	142.0111	146.0518	148.4979	150.0977
118.5	125.9852	127.4624	129.754	133.6295	138.0032	142.4484	146.5103	148.9689	150.5767
119.5	126.3272	127.8132	130.1183	134.0161	138.4143	142.8835	146.9668	149.438	151.0539
120.5	126.6678	128.1625	130.4809	134.4008	138.8234	143.3168	147.4214	149.9053	151.5294
121.5	127.0073	128.5106	130.8422	134.7841	139.231	143.7484	147.8747	150.3714	152.0038
122.5	127.3462	128.8579	131.2026	135.1663	139.6373	144.1789	148.3268	150.8365	152.4773
123.5	127.6851	129.2051	131.5625	135.5477	140.0427	144.6085	148.7782	151.301	152.9504
124.5	128.0243	129.5524	131.9224	135.9288	140.4477	145.0377	149.2294	151.7655	153.4235
125.5	128.3643	129.9004	132.2828	136.3101	140.8527	145.4669	149.6808	152.2303	153.8972
126.5	128.7058	130.2496	132.6441	136.692	141.2582	145.8965	150.1329	152.696	154.3718
127.5	129.0491	130.6005	133.0068	137.075	141.6646	146.3272	150.5861	153.1631	154.848
128.5	129.3949	130.9536	133.3714	137.4597	142.0725	146.7593	151.041	153.6321	155.3263
129.5	129.7436	131.3094	133.7386	137.8466	142.4824	147.1936	151.4982	154.1035	155.8072
130.5	130.0958	131.6686	134.1089	138.2362	142.8949	147.6305	151.9583	154.578	156.2913
131.5	130.452	132.0316	134.4828	138.6292	143.3107	148.0707	152.4218	155.0562	156.7792
132.5	130.8127	132.399	134.8608	139.0262	143.7304	148.5147	152.8894	155.5386	157.2715
133.5	131.1785	132.7714	135.2437	139.4278	144.1545	148.9633	153.3617	156.0258	157.7688
134.5	131.5498	133.1491	135.6318	139.8346	144.5838	149.4172	153.8394	156.5186	158.2717
135.5	131.9272	133.5329	136.026	140.2472	145.019	149.8769	154.323	157.0174	158.7806
136.5	132.311	133.9232	136.4266	140.6664	145.4607	150.3433	154.8133	157.5229	159.2964
137.5	132.7018	134.3205	136.8343	141.0928	145.9097	150.8169	155.3109	158.0356	159.8193
138.5	133.1	134.7252	137.2496	141.5269	146.3665	151.2984	155.8164	158.5562	160.35
139.5	133.5059	135.1378	137.673	141.9694	146.832	151.7885	156.3303	159.0851	160.889
140.5	133.9199	135.5588	138.105	142.4209	147.3066	152.2878	156.8532	159.6228	161.4365
141.5	134.3423	135.9885	138.5461	142.882	147.7911	152.7969	157.3857	160.1697	161.993
142.5	134.7733	136.4271	138.9968	143.3532	148.2859	153.3164	157.928	160.7262	162.5588
143.5	135.2132	136.8751	139.4573	143.835	148.7917	153.8466	158.4807	161.2924	163.1339
144.5	135.6621	137.3326	139.928	144.3277	149.3088	154.3881	159.0439	161.8686	163.7185
145.5	136.1202	137.7998	140.4091	144.8317	149.8376	154.941	159.6179	162.4549	164.3126
146.5	136.5875	138.2769	140.9009	145.3473	150.3784	155.5056	160.2026	163.0511	164.916
147.5	137.064	138.7638	141.4034	145.8746	150.9313	156.0819	160.7981	163.6571	165.5285
148.5	137.5496	139.2605	141.9167	146.4137	151.4964	156.6699	161.4041	164.2726	166.1497
149.5	138.0442	139.767	142.4407	146.9645	152.0735	157.2694	162.0203	164.8972	166.7791
150.5	138.5477	140.2831	142.9752	147.5269	152.6624	157.88	162.6462	165.5302	167.416
151.5	139.0597	140.8085	143.52	148.1005	153.2627	158.5012	163.2811	166.1711	168.0596
152.5	139.5799	141.3429	144.0746	148.6849	153.8738	159.1324	163.9243	166.8187	168.7091
153.5	140.108	141.8859	144.6388	149.2795	154.4951	159.7725	164.5748	167.4723	169.3634
154.5	140.6435	142.4369	145.2117	149.8836	155.1255	160.4207	165.2314	168.1305	170.0213
155.5	141.1858	142.9955	145.7928	150.4962	155.7642	161.0758	165.893	168.7923	170.6817
156.5	141.7345	143.5608	146.3813	151.1165	156.4099	161.7364	166.5581	169.4561	171.343
157.5	142.2889	144.1322	146.9763	151.7433	157.0612	162.401	167.2253	170.1205	172.004
158.5	142.8482	144.7089	147.5767	152.3754	157.7168	163.0682	167.8929	170.784	172.663
159.5	143.4118	145.29	148.1815	153.0113	158.3751	163.7363	168.5594	171.445	173.3186
160.5	143.9788	145.8746	148.7896	153.6498	159.0344	164.4035	169.2231	172.1018	173.9691
161.5	144.5483	146.4615	149.3998	154.2892	159.6931	165.0681	169.8822	172.7528	174.6131
162.5	145.1196	147.0498	150.0107	154.928	160.3493	165.7283	170.535	173.3965	175.249
163.5	145.6915	147.6385	150.621	155.5647	161.0015	166.3823	171.1798	174.0312	175.8753
164.5	146.2633	148.2262	151.2295	156.1977	161.6478	167.0284	171.8151	174.6554	176.4906
165.5	146.8339	148.812	151.8348	156.8253	162.2865	167.665	172.4393	175.2677	177.0935
166.5	147.4023	149.3947	152.4355	157.4462	162.9161	168.2905	173.0509	175.8668	177.6829
167.5	147.9674	149.9731	153.0304	158.0587	163.535	168.9033	173.6486	176.4515	178.2575
168.5	148.5284	150.5461	153.6181	158.6615	164.1418	169.5022	174.2313	177.0206	178.8165
169.5	149.0842	151.1127	154.1975	159.2532	164.7352	170.0859	174.7978	177.5733	179.3589
170.5	149.6338	151.6717	154.7674	159.8327	165.314	170.6535	175.3473	178.1088	179.884
171.5	150.1763	152.2221	155.3268	160.3988	165.8771	171.2039	175.879	178.6264	180.3913
172.5	150.7107	152.763	155.8746	160.9506	166.4236	171.7364	176.3923	179.1256	180.8804
173.5	151.2363	153.2935	156.4099	161.4872	166.9528	172.2504	176.8868	179.6061	181.3509
174.5	151.7521	153.8127	156.9319	162.0078	167.4641	172.7455	177.3622	180.0676	181.8027
175.5	152.2575	154.32	157.4399	162.5118	167.9571	173.2213	177.8183	180.5102	182.2358
176.5	152.7517	154.8147	157.9334	162.9988	168.4313	173.6778	178.2551	180.9338	182.6503
177.5	153.2342	155.2961	158.4118	163.4685	168.8867	174.1148	178.6727	181.3385	183.0463
178.5	153.7043	155.7638	158.8747	163.9205	169.3231	174.5324	179.0712	181.7247	183.4242
179.5	154.1615	156.2174	159.3218	164.3547	169.7405	174.9309	179.451	182.0927	183.7842
180.5	154.6056	156.6566	159.7529	164.7713	170.1393	175.3105	179.8124	182.4429	184.127
181.5	155.036	157.0811	160.168	165.1701	170.5195	175.6716	180.1559	182.7757	184.4528
182.5	155.4526	157.4907	160.5669	165.5514	170.8815	176.0146	180.482	183.0918	184.7624
183.5	155.8552	157.8853	160.9498	165.9154	171.2257	176.34	180.7912	183.3916	185.0562
184.5	156.2436	158.265	161.3167	166.2625	171.5525	176.6483	181.0841	183.6757	185.3349
185.5	156.6178	158.6298	161.6679	166.5929	171.8626	176.9402	181.3614	183.9449	185.599
186.5	156.9777	158.9798	162.0035	166.9072	172.1563	177.2163	181.6236	184.1997	185.8493
187.5	157.3235	159.315	162.3239	167.2057	172.4343	177.4771	181.8715	184.4408	186.0863
188.5	157.6551	159.6359	162.6294	167.489	172.6972	177.7234	182.1056	184.6687	186.3107
189.5	157.9729	159.9425	162.9204	167.7576	172.9456	177.9558	182.3267	184.8843	186.5231
190.5	158.277	160.2352	163.1973	168.012	173.1801	178.175	182.5353	185.0879	186.724
191.5	158.5676	160.5143	163.4605	168.2528	173.4014	178.3815	182.7322	185.2804	186.9142
192.5	158.845	160.7802	163.7104	168.4805	173.6101	178.5762	182.9179	185.4623	187.0941
193.5	159.1095	161.0332	163.9476	168.6958	173.8067	178.7595	183.0931	185.6341	187.2643
194.5	159.3614	161.2738	164.1725	168.8991	173.992	178.9321	183.2583	185.7965	187.4254
195.5	159.6011	161.5023	164.3856	169.0911	174.1665	179.0946	183.414	185.9498	187.5779
196.5	159.829	161.7191	164.5873	169.2722	174.3308	179.2476	183.5609	186.0948	187.7222
197.5	160.0455	161.9247	164.7782	169.4431	174.4854	179.3915	183.6995	186.2318	187.8588
198.5	160.2508	162.1196	164.9587	169.6041	174.631	179.5271	183.8302	186.3613	187.9881
199.5	160.4456	162.3041	165.1292	169.756	174.768	179.6547	183.9535	186.4837	188.1106
200.5	160.63	162.4786	165.2903	169.8991	174.8969	179.7748	184.0699	186.5995	188.2267
201.5	160.8046	162.6437	165.4424	170.0339	175.0182	179.888	184.1797	186.7091	188.3368
202.5	160.9697	162.7997	165.586	170.1608	175.1323	179.9946	184.2835	186.8128	188.4411
203.5	161.1258	162.947	165.7214	170.2804	175.2398	180.095	184.3815	186.911	188.54
204.5	161.2733	163.086	165.8491	170.3931	175.341	180.1896	184.4741	187.004	188.6338
205.5	161.4125	163.2172	165.9694	170.4991	175.4362	180.2789	184.5617	187.0922	188.7229
206.5	161.5438	163.3409	166.0828	170.599	175.5259	180.3631	184.6446	187.1757	188.8075
207.5	161.6676	163.4575	166.1897	170.693	175.6104	180.4426	184.723	187.255	188.8878
208.5	161.7843	163.5673	166.2903	170.7816	175.6901	180.5176	184.7972	187.3302	188.9642
209.5	161.8942	163.6708	166.3851	170.865	175.7652	180.5885	184.8676	187.4016	189.0368
210.5	161.9977	163.7682	166.4743	170.9436	175.836	180.6555	184.9343	187.4694	189.1058
211.5	162.0951	163.8598	166.5583	171.0176	175.9028	180.7189	184.9975	187.5338	189.1715
212.5	162.1866	163.9461	166.6373	171.0873	175.9658	180.7789	185.0576	187.5951	189.234
213.5	162.2727	164.0272	166.7116	171.1529	176.0254	180.8357	185.1146	187.6534	189.2936
214.5	162.3537	164.1034	166.7816	171.2148	176.0816	180.8895	185.1687	187.7088	189.3503
215.5	162.4297	164.1751	166.8474	171.2732	176.1348	180.9405	185.2202	187.7617	189.4044
216.5	162.5011	164.2424	166.9094	171.3282	176.185	180.9889	185.2692	187.812	189.456
217.5	162.5681	164.3057	166.9676	171.3801	176.2326	181.0348	185.3159	187.86	189.5052
218.5	162.631	164.3651	167.0224	171.429	176.2776	181.0784	185.3603	187.9057	189.5522
219.5	162.69	164.4209	167.074	171.4752	176.3202	181.1199	185.4026	187.9494	189.5971
220.5	162.7453	164.4733	167.1224	171.5188	176.3606	181.1593	185.443	187.9911	189.6399
221.5	162.7972	164.5224	167.168	171.5599	176.3989	181.1968	185.4815	188.0309	189.6809
222.5	162.8458	164.5686	167.2109	171.5988	176.4352	181.2325	185.5182	188.069	189.7201
223.5	162.8914	164.6119	167.2513	171.6355	176.4697	181.2666	185.5534	188.1054	189.7575
224.5	162.9341	164.6526	167.2892	171.6701	176.5024	181.299	185.5869	188.1402	189.7934
225.5	162.9741	164.6907	167.325	171.7029	176.5335	181.33	185.619	188.1736	189.8277
226.5	163.0115	164.7265	167.3585	171.7339	176.563	181.3595	185.6497	188.2055	189.8606
227.5	163.0465	164.76	167.3902	171.7632	176.5911	181.3877	185.6791	188.236	189.8922
228.5	163.0793	164.7915	167.4199	171.791	176.6179	181.4147	185.7073	188.2653	189.9224
229.5	163.11	164.821	167.4479	171.8172	176.6433	181.4405	185.7343	188.2934	189.9513
230.5	163.1387	164.8487	167.4742	171.8421	176.6676	181.4651	185.7601	188.3204	189.9791
231.5	163.1656	164.8746	167.499	171.8657	176.6907	181.4887	185.7849	188.3462	190.0058
232.5	163.1907	164.8989	167.5224	171.888	176.7127	181.5113	185.8087	188.3711	190.0314
233.5	163.2142	164.9217	167.5444	171.9091	176.7337	181.533	185.8316	188.3949	190.056
234.5	163.2361	164.9431	167.5651	171.9292	176.7538	181.5538	185.8535	188.4178	190.0797
235.5	163.2566	164.9631	167.5846	171.9483	176.773	181.5737	185.8746	188.4399	190.1024
236.5	163.2757	164.9819	167.6029	171.9663	176.7913	181.5928	185.8949	188.461	190.1242
237.5	163.2936	164.9995	167.6203	171.9835	176.8088	181.6111	185.9144	188.4814	190.1452
238.5	163.3103	165.016	167.6366	171.9998	176.8255	181.6287	185.9331	188.501	190.1654
239.5	163.3259	165.0315	167.6519	172.0153	176.8415	181.6456	185.9512	188.5198	190.1849
240	163.3333	165.0389	167.6593	172.0227	176.8492	181.6538	185.9599	188.529	190.1943

Appendix B 

**Table 10:** Data table of stature-for-age charts (females, age 2–20 years) as per CDC guidelines [12]

Age (in months)	3rd Percentile Stature (in centimeters)	5th Percentile Stature (in centimeters)	10th Percentile Stature (in centimeters)	25th Percentile Stature (in centimeters)	50th Percentile Stature (in centimeters)	75th Percentile Stature (in centimeters)	90th Percentile Stature (in centimeters)	95th Percentile Stature (in centimeters)	97th Percentile Stature (in centimeters)
24	78.43754	79.25982	80.52476	82.63524	84.97556	87.31121	89.40951	90.66355	91.47729
24.5	78.82133	79.64777	80.91946	83.04213	85.39732	87.74918	89.86316	91.12707	91.94741
25.5	79.60198	80.44226	81.73541	83.8943	86.29026	88.68344	90.83505	92.12168	92.95685
26.5	80.37555	81.22666	82.53699	84.72592	87.15714	89.58751	91.77421	93.08254	93.93209
27.5	81.1357	81.9954	83.31968	85.53389	87.99602	90.46018	92.67969	94.00873	94.87215
28.5	81.87746	82.74411	84.07998	86.31589	88.80551	91.30065	93.55097	94.89974	95.77649
29.5	82.59712	83.46957	84.81532	87.07028	89.58477	92.10859	94.38793	95.75551	96.64505
30.5	83.29206	84.16953	85.52398	87.79609	90.33342	92.88403	95.19083	96.57635	97.47814
31.5	83.96065	84.84264	86.205	88.49291	91.05154	93.62741	95.9603	97.36295	98.27646
32.5	84.6021	85.4883	86.85807	89.16084	91.73964	94.33951	96.69729	98.11632	99.04107
33.5	85.2163	86.10656	87.48344	89.80045	92.39854	95.0214	97.40303	98.83778	99.77332
34.5	85.80379	86.69803	88.08186	90.4127	93.02945	95.67446	98.07904	99.52891	100.4748
35.5	86.36557	87.26379	88.6545	90.99891	93.63382	96.30029	98.72705	100.1915	101.1474
36.5	86.90307	87.80528	89.20285	91.56066	94.21336	96.90071	99.34899	100.8276	101.7931
37.5	87.43482	88.34236	89.74875	92.12298	94.79643	97.50724	99.97896	101.4726	102.4485
38.5	87.95945	88.87256	90.28811	92.67925	95.37392	98.10855	100.604	102.1129	103.0991
39.5	88.4785	89.39733	90.82228	93.2307	95.94693	98.70568	101.2251	102.7494	103.746
40.5	88.9933	89.91797	91.35246	93.7784	96.51645	99.29957	101.8432	103.383	104.3901
41.5	89.50502	90.43559	91.87972	94.32334	97.08337	99.89104	102.459	104.0144	105.032
42.5	90.01466	90.95115	92.40497	94.86634	97.64848	100.4808	103.0732	104.6444	105.6727
43.5	90.52307	91.46549	92.92901	95.40817	98.21247	101.0696	103.6866	105.2736	106.3126
44.5	91.031	91.97932	93.45252	95.94946	98.77593	101.6579	104.2996	105.9025	106.9523
45.5	91.53905	92.49325	93.97609	96.49076	99.3394	102.2462	104.9128	106.5316	107.5922
46.5	92.04774	93.00778	94.50021	97.03254	99.90331	102.835	105.5264	107.1613	108.2328
47.5	92.55748	93.52333	95.02528	97.57519	100.4681	103.4247	106.141	107.7919	108.8744
48.5	93.06862	94.04022	95.55164	98.11905	101.0339	104.0154	106.7567	108.4238	109.5172
49.5	93.58141	94.55872	96.07954	98.66436	101.6012	104.6075	107.3737	109.057	110.1614
50.5	94.09605	95.07903	96.60918	99.21132	102.17	105.2012	107.9924	109.6918	110.8073
51.5	94.61267	95.60128	97.14072	99.76009	102.7406	105.7965	108.6127	110.3283	111.4548
52.5	95.13134	96.12555	97.67423	100.3108	103.313	106.3936	109.2347	110.9665	112.1041
53.5	95.65211	96.65189	98.20976	100.8634	103.8873	106.9925	109.8585	111.6066	112.7552
54.5	96.17495	97.18029	98.74731	101.418	104.4635	107.5933	110.4841	112.2483	113.4079
55.5	96.69982	97.71069	99.28686	101.9745	105.0415	108.1958	111.1114	112.8917	114.0624
56.5	97.22663	98.24303	99.82832	102.5329	105.6213	108.8001	111.7404	113.5368	114.7184
57.5	97.75525	98.77719	100.3716	103.093	106.2029	109.406	112.3709	114.1833	115.3759
58.5	98.28555	99.31303	100.9165	103.6549	106.7861	110.0134	113.0028	114.8312	116.0347
59.5	98.81735	99.85039	101.463	104.2182	107.3707	110.6222	113.6359	115.4802	116.6945
60.5	99.35047	100.3891	102.0109	104.7829	107.9566	111.2321	114.2701	116.1301	117.3552
61.5	99.8847	100.9289	102.5599	105.3488	108.5436	111.8431	114.9052	116.7808	118.0166
62.5	100.4198	101.4696	103.1098	105.9156	109.1316	112.4548	115.5408	117.432	118.6783
63.5	100.9555	102.011	103.6604	106.4831	109.7202	113.0671	116.1768	118.0834	119.3402
64.5	101.4916	102.5529	104.2115	107.0512	110.3092	113.6797	116.813	118.7348	120.0019
65.5	102.0279	103.0948	104.7628	107.6194	110.8984	114.2923	117.449	119.3858	120.6632
66.5	102.564	103.6367	105.3141	108.1877	111.4876	114.9048	118.0845	120.0362	121.3238
67.5	103.0996	104.1782	105.865	108.7556	112.0764	115.5167	118.7193	120.6857	121.9832
68.5	103.6346	104.7191	106.4154	109.323	112.6646	116.1278	119.3531	121.334	122.6413
69.5	104.1685	105.259	106.9648	109.8895	113.2519	116.7379	119.9855	121.9807	123.2977
70.5	104.7012	105.7976	107.5131	110.4549	113.838	117.3466	120.6163	122.6256	123.9521
71.5	105.2323	106.3348	108.0599	111.0189	114.4226	117.9537	121.2452	123.2684	124.6042
72.5	105.7615	106.8701	108.605	111.5812	115.0055	118.5588	121.8718	123.9086	125.2536
73.5	106.2886	107.4033	109.148	112.1415	115.5863	119.1616	122.4959	124.5461	125.9
74.5	106.8132	107.9342	109.6888	112.6996	116.1648	119.7619	123.1171	125.1804	126.5432
75.5	107.3351	108.4624	110.227	113.255	116.7406	120.3594	123.7352	125.8114	127.1827
76.5	107.8541	108.9877	110.7623	113.8077	117.3136	120.9537	124.3499	126.4387	127.8184
77.5	108.3698	109.5099	111.2944	114.3572	117.8833	121.5447	124.9608	127.062	128.45
78.5	108.882	110.0285	111.8232	114.9034	118.4496	122.132	125.5678	127.6811	129.0771
79.5	109.3905	110.5435	112.3483	115.446	119.0123	122.7154	126.1705	128.2957	129.6996
80.5	109.8949	111.0545	112.8696	115.9847	119.571	123.2946	126.7688	128.9056	130.3171
81.5	110.3952	111.5613	113.3867	116.5193	120.1254	123.8695	127.3623	129.5105	130.9295
82.5	110.8909	112.0638	113.8995	117.0496	120.6755	124.4397	127.951	130.1103	131.5365
83.5	111.3821	112.5616	114.4077	117.5754	121.221	125.0051	128.5345	130.7047	132.138
84.5	111.8684	113.0546	114.9112	118.0964	121.7617	125.5655	129.1127	131.2936	132.7338
85.5	112.3496	113.5427	115.4097	118.6125	122.2974	126.1207	129.6855	131.8768	133.3238
86.5	112.8257	114.0256	115.9031	119.1235	122.8279	126.6706	130.2526	132.4542	133.9077
87.5	113.2963	114.5031	116.3913	119.6293	123.3531	127.215	130.814	133.0256	134.4857
88.5	113.7615	114.9752	116.874	120.1297	123.8728	127.7539	131.3696	133.5911	135.0574
89.5	114.2211	115.4418	117.3512	120.6246	124.387	128.287	131.9194	134.1505	135.623
90.5	114.6749	115.9026	117.8228	121.1138	124.8956	128.8144	132.4631	134.7038	136.1824
91.5	115.123	116.3577	118.2886	121.5974	125.3985	129.3359	133.0009	135.251	136.7356
92.5	115.5651	116.8069	118.7486	122.0753	125.8956	129.8516	133.5328	135.7922	137.2826
93.5	116.0012	117.2502	119.2028	122.5473	126.3869	130.3615	134.0587	136.3273	137.8236
94.5	116.4314	117.6875	119.6511	123.0135	126.8724	130.8656	134.5787	136.8565	138.3585
95.5	116.8555	118.1189	120.0935	123.4739	127.3522	131.364	135.093	137.3798	138.8876
96.5	117.2737	118.5443	120.53	123.9285	127.8263	131.8567	135.6015	137.8975	139.411
97.5	117.6858	118.9638	120.9607	124.3774	128.2947	132.3438	136.1046	138.4097	139.9289
98.5	118.092	119.3774	121.3855	124.8207	128.7576	132.8255	136.6024	138.9166	140.4415
99.5	118.4924	119.7852	121.8047	125.2584	129.2152	133.302	137.095	139.4184	140.9492
100.5	118.8869	120.1873	122.2182	125.6906	129.6675	133.7734	137.5828	139.9155	141.4521
101.5	119.2757	120.5838	122.6263	126.1177	130.1148	134.2401	138.066	140.4082	141.9507
102.5	119.659	120.9748	123.0291	126.5396	130.5574	134.7023	138.545	140.8968	142.4454
103.5	120.037	121.3606	123.4268	126.9568	130.9954	135.1604	139.0201	141.3817	142.9364
104.5	120.4097	121.7413	123.8196	127.3694	131.4293	135.6146	139.4918	141.8633	143.4244
105.5	120.7775	122.1171	124.2078	127.7777	131.8593	136.0654	139.9604	142.3422	143.9098
106.5	121.1405	122.4884	124.5916	128.1822	132.2859	136.5132	140.4265	142.8188	144.393
107.5	121.4991	122.8555	124.9715	128.5831	132.7094	136.9585	140.8906	143.2937	144.8747
108.5	121.8537	123.2186	125.3478	128.9808	133.1304	137.4018	141.3532	143.7674	145.3555
109.5	122.2044	123.5782	125.7208	129.3759	133.5493	137.8437	141.8149	144.2406	145.8359
110.5	122.5518	123.9347	126.0911	129.7689	133.9667	138.2847	142.2764	144.7139	146.3167
111.5	122.8963	124.2885	126.4592	130.1603	134.3832	138.7256	142.7382	145.1879	146.7984
112.5	123.2384	124.6402	126.8255	130.5506	134.7995	139.1669	143.2012	145.6634	147.2818
113.5	123.5785	124.9902	127.1907	130.9406	135.2163	139.6094	143.666	146.141	147.7676
114.5	123.9173	125.3393	127.5554	131.3309	135.6342	140.0538	144.1333	146.6215	148.2564
115.5	124.2553	125.688	127.9203	131.7223	136.054	140.501	144.6039	147.1056	148.7491
116.5	124.5933	126.0371	128.2861	132.1156	136.4766	140.9516	145.0785	147.594	149.2461
117.5	124.932	126.3872	128.6537	132.5115	136.9027	141.4065	145.5579	148.0874	149.7484
118.5	125.2721	126.7392	129.0238	132.9109	137.3333	141.8665	146.0429	148.5865	150.2564
119.5	125.6144	127.094	129.3973	133.3147	137.7691	142.3324	146.5341	149.092	150.7707
120.5	125.9599	127.4524	129.7752	133.7239	138.2112	142.8051	147.0322	149.6044	151.292
121.5	126.3095	127.8154	130.1584	134.1394	138.6602	143.2852	147.5379	150.1242	151.8205
122.5	126.6641	128.184	130.5479	134.562	139.1172	143.7735	148.0517	150.652	152.3568
123.5	127.0248	128.5591	130.9446	134.9929	139.5829	144.2707	148.5741	151.188	152.9011
124.5	127.3926	128.9419	131.3496	135.4328	140.0581	144.7773	149.1054	151.7325	153.4534
125.5	127.7687	129.3334	131.7639	135.8826	140.5435	145.2938	149.646	152.2856	154.0139
126.5	128.1541	129.7346	132.1885	136.3433	141.0397	145.8206	150.196	152.8473	154.5824
127.5	128.5499	130.1467	132.6243	136.8154	141.5472	146.3579	150.7552	153.4174	155.1586
128.5	128.9573	130.5705	133.0721	137.2997	142.0664	146.9059	151.3236	153.9955	155.742
129.5	129.3772	131.0071	133.5329	137.7967	142.5974	147.4643	151.9008	154.5812	156.3321
130.5	129.8106	131.4573	134.0072	138.3067	143.1404	148.0329	152.4861	155.1737	156.928
131.5	130.2585	131.9218	134.4955	138.83	143.695	148.6111	153.079	155.7721	157.5288
132.5	130.7217	132.4013	134.9983	139.3664	144.2609	149.1984	153.6783	156.3755	158.1335
133.5	131.2006	132.8962	135.5157	139.9157	144.8376	149.7937	154.283	156.9825	158.7407
134.5	131.6958	133.4067	136.0476	140.4775	145.424	150.3959	154.8918	157.5918	159.3491
135.5	132.2074	133.9328	136.5937	141.051	146.0192	151.0036	155.5032	158.202	159.9571
136.5	132.7354	134.4742	137.1534	141.6352	146.6217	151.6153	156.1156	158.8115	160.5633
137.5	133.2795	135.0304	137.7259	142.2288	147.23	152.2293	156.7273	159.4185	161.166
138.5	133.8388	135.6004	138.31	142.8304	147.8424	152.8438	157.3365	160.0213	161.7634
139.5	134.4125	136.1831	138.9043	143.4381	148.4569	153.4568	157.9413	160.6182	162.3541
140.5	134.9993	136.7769	139.507	144.0501	149.0714	154.0662	158.5398	161.2075	162.9363
141.5	135.5973	137.3801	140.1161	144.6641	149.6839	154.67	159.1302	161.7874	163.5084
142.5	136.2047	137.9905	140.7295	145.278	150.292	155.2663	159.7107	162.3564	164.069
143.5	136.8191	138.6058	141.3448	145.8893	150.8936	155.8529	160.2796	162.9129	164.6167
144.5	137.4381	139.2236	141.9594	146.4958	151.4866	156.428	160.8353	163.4555	165.1503
145.5	138.0588	139.841	142.5709	147.0949	152.0687	156.9899	161.3764	163.983	165.6685
146.5	138.6784	140.4554	143.1767	147.6845	152.6381	157.5369	161.9016	164.4943	166.1706
147.5	139.2941	141.064	143.7741	148.2623	153.193	158.0677	162.4097	164.9885	166.6555
148.5	139.9028	141.6641	144.3607	148.8263	153.7317	158.581	162.8999	165.4648	167.1228
149.5	140.5019	142.253	144.9342	149.3747	154.2529	159.0758	163.3715	165.9227	167.572
150.5	141.0885	142.8283	145.4925	149.9059	154.7555	159.5513	163.8239	166.3618	168.0027
151.5	141.6602	143.3877	146.0338	150.4184	155.2385	160.007	164.2568	166.7819	168.4147
152.5	142.2148	143.9294	146.5564	150.9113	155.7012	160.4425	164.6701	167.1829	168.808
153.5	142.7504	144.4516	147.059	151.3835	156.1432	160.8576	165.0637	167.5648	169.1827
154.5	143.2654	144.953	147.5405	151.8346	156.5643	161.2524	165.4378	167.9278	169.5391
155.5	143.7584	145.4325	148.0002	152.2642	156.9644	161.627	165.7928	168.2723	169.8773
156.5	144.2287	145.8894	148.4376	152.6721	157.3437	161.9818	166.1289	168.5987	170.1979
157.5	144.6756	146.3232	148.8525	153.0584	157.7025	162.3172	166.4466	168.9074	170.5013
158.5	145.0987	146.7338	149.2449	153.4234	158.0411	162.6338	166.7467	169.199	170.7881
159.5	145.4981	147.1213	149.615	153.7674	158.3603	162.9321	167.0296	169.4742	171.0587
160.5	145.874	147.4859	149.9633	154.0911	158.6606	163.2129	167.2961	169.7335	171.314
161.5	146.2269	147.8281	150.2902	154.3951	158.9427	163.477	167.5469	169.9777	171.5544
162.5	146.5573	148.1487	150.5966	154.6801	159.2075	163.725	167.7826	170.2074	171.7807
163.5	146.866	148.4483	150.8831	154.947	159.4557	163.9577	168.0042	170.4234	171.9935
164.5	147.1539	148.7279	151.1507	155.1966	159.6882	164.1761	168.2122	170.6263	172.1936
165.5	147.4219	148.9885	151.4003	155.4298	159.9058	164.3808	168.4075	170.817	172.3816
166.5	147.6712	149.2309	151.6329	155.6475	160.1094	164.5726	168.5907	170.9959	172.5582
167.5	147.9026	149.4562	151.8494	155.8507	160.2997	164.7523	168.7626	171.1639	172.7239
168.5	148.1173	149.6655	152.0508	156.0401	160.4777	164.9206	168.9239	171.3216	172.8796
169.5	148.3164	149.8598	152.2381	156.2167	160.6441	165.0783	169.0751	171.4696	173.0257
170.5	148.5009	150.04	152.4121	156.3813	160.7995	165.226	169.217	171.6085	173.1628
171.5	148.6717	150.2072	152.5738	156.5348	160.9449	165.3644	169.3501	171.7388	173.2915
172.5	148.8299	150.3621	152.7241	156.6778	161.0808	165.4941	169.4749	171.8611	173.4124
173.5	148.9764	150.5059	152.8638	156.8112	161.2079	165.6157	169.5921	171.976	173.5258
174.5	149.1121	150.6392	152.9936	156.9356	161.3268	165.7297	169.7022	172.0839	173.6324
175.5	149.2377	150.7629	153.1143	157.0517	161.4381	165.8366	169.8055	172.1853	173.7326
176.5	149.3542	150.8777	153.2266	157.16	161.5423	165.9369	169.9026	172.2806	173.8267
177.5	149.4622	150.9843	153.3312	157.2612	161.6399	166.0312	169.9939	172.3701	173.9152
178.5	149.5623	151.0833	153.4286	157.3558	161.7315	166.1197	170.0798	172.4544	173.9984
179.5	149.6553	151.1754	153.5193	157.4443	161.8174	166.2029	170.1606	172.5337	174.0768
180.5	149.7416	151.2611	153.604	157.5271	161.898	166.2812	170.2366	172.6084	174.1505
181.5	149.8219	151.341	153.683	157.6047	161.9738	166.3549	170.3083	172.6787	174.22
182.5	149.8967	151.4154	153.7569	157.6775	162.045	166.4244	170.3759	172.7451	174.2855
183.5	149.9663	151.4848	153.826	157.7458	162.112	166.4898	170.4396	172.8076	174.3472
184.5	150.0312	151.5497	153.8907	157.8099	162.1752	166.5516	170.4997	172.8667	174.4055
185.5	150.0918	151.6103	153.9513	157.8702	162.2347	166.6099	170.5566	172.9225	174.4606
186.5	150.1484	151.6671	154.0082	157.927	162.2908	166.6649	170.6103	172.9752	174.5125
187.5	150.2014	151.7203	154.0616	157.9804	162.3439	166.717	170.6611	173.025	174.5617
188.5	150.251	151.7702	154.1119	158.0308	162.394	166.7663	170.7091	173.0722	174.6082
189.5	150.2975	151.8171	154.1592	158.0784	162.4414	166.8129	170.7546	173.1168	174.6522
190.5	150.3412	151.8612	154.2037	158.1234	162.4862	166.8571	170.7978	173.1591	174.6938
191.5	150.3823	151.9027	154.2457	158.1659	162.5287	166.899	170.8387	173.1992	174.7333
192.5	150.4209	151.9418	154.2854	158.2061	162.569	166.9388	170.8775	173.2373	174.7708
193.5	150.4573	151.9787	154.3229	158.2442	162.6072	166.9766	170.9144	173.2734	174.8063
194.5	150.4917	152.0135	154.3584	158.2803	162.6435	167.0125	170.9494	173.3077	174.84
195.5	150.5241	152.0465	154.3919	158.3146	162.6781	167.0466	170.9827	173.3402	174.8721
196.5	150.5547	152.0776	154.4238	158.3472	162.7109	167.0791	171.0144	173.3712	174.9025
197.5	150.5837	152.1072	154.454	158.3782	162.7421	167.11	171.0446	173.4007	174.9314
198.5	150.6111	152.1352	154.4827	158.4077	162.7719	167.1395	171.0733	173.4288	174.959
199.5	150.6372	152.1617	154.51	158.4357	162.8002	167.1676	171.1007	173.4555	174.9852
200.5	150.6619	152.187	154.5359	158.4625	162.8273	167.1944	171.1268	173.481	175.0102
201.5	150.6854	152.211	154.5607	158.4879	162.8531	167.22	171.1517	173.5053	175.034
202.5	150.7077	152.2339	154.5842	158.5123	162.8778	167.2444	171.1754	173.5284	175.0567
203.5	150.7289	152.2556	154.6067	158.5355	162.9013	167.2677	171.1981	173.5505	175.0783
204.5	150.7491	152.2764	154.6281	158.5577	162.9238	167.29	171.2198	173.5716	175.099
205.5	150.7684	152.2962	154.6486	158.5789	162.9454	167.3114	171.2405	173.5918	175.1187
206.5	150.7868	152.3151	154.6681	158.5992	162.966	167.3318	171.2604	173.6111	175.1376
207.5	150.8044	152.3332	154.6868	158.6187	162.9858	167.3514	171.2793	173.6295	175.1556
208.5	150.8211	152.3504	154.7047	158.6373	163.0047	167.3701	171.2975	173.6471	175.1728
209.5	150.8372	152.3669	154.7218	158.6551	163.0228	167.3881	171.3149	173.664	175.1892
210.5	150.8525	152.3827	154.7382	158.6722	163.0402	167.4053	171.3315	173.6802	175.205
211.5	150.8672	152.3979	154.754	158.6886	163.0569	167.4218	171.3475	173.6956	175.2201
212.5	150.8812	152.4124	154.769	158.7043	163.0729	167.4376	171.3628	173.7104	175.2345
213.5	150.8947	152.4263	154.7835	158.7194	163.0882	167.4528	171.3775	173.7246	175.2483
214.5	150.9076	152.4396	154.7974	158.7339	163.103	167.4674	171.3915	173.7382	175.2616
215.5	150.92	152.4524	154.8107	158.7478	163.1172	167.4814	171.405	173.7513	175.2742
216.5	150.9319	152.4647	154.8235	158.7612	163.1308	167.4948	171.418	173.7638	175.2864
217.5	150.9433	152.4765	154.8358	158.774	163.1439	167.5078	171.4304	173.7758	175.2981
218.5	150.9542	152.4878	154.8476	158.7864	163.1565	167.5202	171.4424	173.7873	175.3093
219.5	150.9647	152.4987	154.859	158.7983	163.1686	167.5321	171.4538	173.7984	175.32
220.5	150.9749	152.5092	154.8699	158.8097	163.1802	167.5436	171.4649	173.809	175.3303
221.5	150.9846	152.5192	154.8804	158.8207	163.1914	167.5546	171.4755	173.8192	175.3402
222.5	150.9939	152.5289	154.8905	158.8313	163.2022	167.5653	171.4856	173.829	175.3497
223.5	151.0029	152.5382	154.9003	158.8415	163.2126	167.5755	171.4954	173.8384	175.3588
224.5	151.0115	152.5472	154.9096	158.8514	163.2226	167.5853	171.5049	173.8474	175.3675
225.5	151.0198	152.5558	154.9187	158.8608	163.2322	167.5948	171.5139	173.8561	175.376
226.5	151.0279	152.5641	154.9273	158.8699	163.2415	167.6039	171.5226	173.8645	175.384
227.5	151.0356	152.5721	154.9357	158.8787	163.2504	167.6127	171.531	173.8725	175.3918
228.5	151.043	152.5798	154.9438	158.8872	163.259	167.6211	171.5391	173.8802	175.3993
229.5	151.0501	152.5873	154.9516	158.8953	163.2673	167.6293	171.5468	173.8877	175.4064
230.5	151.057	152.5944	154.959	158.9032	163.2753	167.6371	171.5543	173.8948	175.4133
231.5	151.0636	152.6013	154.9663	158.9107	163.283	167.6446	171.5615	173.9017	175.42
232.5	151.07	152.6079	154.9732	158.918	163.2904	167.6519	171.5684	173.9083	175.4264
233.5	151.0762	152.6143	154.9799	158.9251	163.2976	167.6589	171.5751	173.9147	175.4325
234.5	151.0821	152.6205	154.9864	158.9319	163.3045	167.6657	171.5815	173.9208	175.4384
235.5	151.0879	152.6265	154.9926	158.9384	163.3111	167.6722	171.5877	173.9267	175.4441
236.5	151.0934	152.6322	154.9986	158.9447	163.3175	167.6785	171.5937	173.9324	175.4496
237.5	151.0987	152.6377	155.0044	158.9508	163.3237	167.6845	171.5994	173.9379	175.4548
238.5	151.1038	152.6431	155.01	158.9567	163.3297	167.6904	171.6049	173.9432	175.4599
239.5	151.1088	152.6482	155.0154	158.9624	163.3354	167.696	171.6103	173.9482	175.4648
240	151.1112	152.6507	155.0181	158.9651	163.3383	167.6987	171.6129	173.9507	175.4671
